# DYNAMICS OF DAILY LONELINESS AND DIURNAL CORTISOL

**DOI:** 10.1093/geroni/igae098.1880

**Published:** 2024-12-31

**Authors:** Karina Van Bogart, David Almeida, John Felt, Jonathan Rush, Eric Cerino, Susan Charles

**Affiliations:** Northwestern University, Chicago, Illinois, United States; Pennsylvania State University, University Park, Pennsylvania, United States; The Pennsylvania State University, University Park, Pennsylvania, United States; University of Victoria, Victoria, British Columbia, Canada; Northern Arizona University, Flagstaff, Arizona, United States; University of California Irvine, Irvine, California, United States

## Abstract

Loneliness impedes healthy aging. Previous research shows robust findings between loneliness and poor health, including stress-related diseases and conditions (e.g., cardiovascular disease). Less is known about how loneliness experienced in daily life relates to indices of health, such as diurnal cortisol level patterns. The slope of diurnal cortisol shows a peak upon waking from sleep and a nadir close to bedtime; however, specific rhythms of cortisol slopes fluctuate in daily life. Emerging research suggests that capturing the dynamic range of diurnal cortisol in daily life could be an important marker of allostatic load (i.e., the cumulative burden of chronic stress), with a compressed range indicating dysregulation and a wider range suggesting a more adaptive cortisol profile. In the current research, we investigated whether daily self-reported loneliness related to the dynamic range of diurnal cortisol (calculated via 4 days of salivary cortisol collection, 4 times a day) and explored age differences across the lifespan. Data come from a national daily assessment study with participants ranging between 25-75 years old who completed 8 days of daily diary assessments on loneliness and other factors in daily life. Findings from the current analyses will be presented in the context of how the dynamic range of cortisol might serve as a potential mechanism for outcomes related to loneliness in later life.

